# Bibliographical Notices

**Published:** 1854-04

**Authors:** 


### BIBLIOGRAPHICAL NOTICES.

*Pneumonia : Its supposed connection, Pathological and Etiological, with Autumnal Fevers : including an Inquiry into the Existence and Morbid Agency of Malaria.* By R. La Roche, M. D., Member of the American Philosophical Society ; of the American Medical Association ; Fellow of the College of Physicians of Philadelphia ; Corresponding Member of the Imperial Academy of Medicine, and Foreign Associate of the Medical Society of Emulation of Paris ; of the Academies of Science at Turin, Copenhagen, Stockholm and Nancy ; and of the Medical Societies of Marseilles, Lyons, &c., &c.

This work is a valuable contribution to medical literature, befitting the importance of the subject investigated and the character of the author for patient and extended researches, made in a conscientious and truth-seeking spirit. It places before the reader not only the opinions held on marsh exhalations and malaria in general, from an early period down to the present day, but also a large and well marshalled array of facts in support of the popular belief of the existence of a subtle poison, which causes periodical fevers, in a fearfully large number of those who are exposed to its operations. We ought not to be obliged to insist on the importance of inquiries of this nature, in a practical point of view ; since a knowledge of the cause of a disease, though it may not suggest a remedy, has higher claims even than this, in its pointing out the means of prevention, and thus bringing our philosophy to bear on the masses, in place of uncertain dealings with individuals. But we are afraid that this natural induction from studies in etiology does not present itself readily to the minds of a majority of our medical brethren, too many of whom would narrow our noble science into a collection of empirical dicta, under the title of practical medicine, and impliedly, if not by direct proscription, consign to oblivion its rich literature, the history of all former times, with its encouragements and warnings, its records of great truths, and grave judgment on various heresies of doctrine. Such opinions coming from the illiterate vulgar need not excite surprise ; but when uttered by members of a liberal profession must induce fears of a decline of learning and an abandonment of the exercise of those higher faculties which distinguish man from the animal mechanic, that builds its house after one invariable style, knowing no change and seeking none. The beaver, the ant, and the bee are very practical bodies; and if they had some of our disparagers of learning and progress to be exponents of their opinions, they would, doubtless, pity a Vitruvius, a Palladio, a Bramante, and a Wren, as speculative enthusiasts filled with vain theories on the construction and arrangement of edifices, which should combine convenience with ornament, majestic proportions with beauty of detail, and exhibit the greatest variety of adaptation to man's use for the worship of his Creator, the meeting of national and corporate councils, and the gratification of his tastes by the introduction of the fine arts, and of his comfort in domestic life by appropriate architectural aids.

There are villages in Armenia which consist of houses made by excavations in the hills, so as to have no entrance except on the side; and others are entirely subterranean, with only small conical apertures in the roof for the admission of a few rays of light and the escape of smoke. "The traveller," says the Rev. Mr. Perkins, the missionary, "often comes upon these villages without any previous notice; and when travelling in the night, his first knowledge of being among human habitations may be, that he finds himself, with the animal he rides, upon the roofs of their houses." These serve both as dwellings for the biped human bodies, and stables for the quadrupeds. The people are mostly of the shepherd class, and live in the same manner as their ancestors, whom Xenophon encountered in the memorable retreat of the "ten thousand," did. They, good practical souls, are no theorists, no book-worms! They are not vexed with cattle shows or agricultural fairs, or any such disturbing exhibitions; nor are their brains perplexed with disquisitions on commerce and political economy in general. The oven in which they bake their bread, serves, also, in conjunction with the breath, and cutaneous exhalations of themselves and their brute companions, to keep them warm; and beyond this, what need they care for such speculative subjects as the functions of respiration and calorification, and the accumulation of carbonic acid in perilous, and it must be, at times, often fatal quantity. They have experience, too, on their side. Did not their fathers, and their grandfathers, and their great-grandfathers, if they trouble themselves with so much historical retrospection, live as they now live, and as they see their children living under their eyes—a practical refutation of all the new-fangled notions of pure air and ventilation, and solar light, being necessary for the enjoyment of full health by the animal creation? As to the comparisons drawn from vital statistics, they must be deemed to be quite superfluous and inapplicable to them. Can they not bear to be sweated, and to be smoked, and to breathe a close, stinking air better than the inhabitants of any above-ground village in the land, and what better test of strength and endurance need they offer? Some will smile at this process of reasoning, and may call it inconclusive; but it is quite as logical as that resorted to by persons who speak lightly, and even sometimes with an air of commiseration of those who are proficients in learning and addicted to literary research, on the plea of the incompatibility of such pursuits with the active and successful discharge of professional or of other duties; as if a knowledge of the difficulties which others, similarly situated as ourselves, encounter, would increase our own; or as if the means by which they achieved success would weaken our efforts to obtain the like pleasant result. Is each successive traveller over a new and imperfectly explored country to refuse to be benefited by the recorded experience and admonitory cautions and reflexions of others who have made the journey, for the reason that he ought to trust entirely to his own observations, lest his resolution would be daunted and his senses enfeebled by his knowing the impressions made on those of others? He is bound, forsooth, if he would not give up his character for independent thought and action, to discover, for himself, how he is to thread his way through a mountain defile, where to ford a river, what precipice to shun, near which his path lies, what spring he may expect to meet in the desert, with whose waters he can slake his thirst and revive his fainting frame. He may, indeed, never reach his journey's end; he may be drowned in the river, or fall headlong over the precipice, never to rise again, or sink down exanimate in the desert; but his friends will, in any of these events, have the proud satisfaction of knowing that he evinced his self-reliance to the last, and died, indebted to no man for information that might have saved his life, and enabled him to return home, and to add his share to the lore accumulated by preceding travellers.

In like manner a young physician, about to enter on the eventful career of the practice of medicine, may be told to rely exclusively on his own observations and resulting experience, to mistrust the language of books, and to avoid becoming an author. He cannot, however, reach sane conclusions in pathology and therapeutics, at the bed side of his first patients, by intuition. He must have had some guidance, if it is only the empirical experience, in dogmatic form, of his first preceptor, and a certain amount of elementary knowledge, seasoned with the condiments of various speculations and theories of his professors, during collegiate periods comprised within, for the most part, the narrow limits of eight months. Add to these a few clinical observations made in the practice of his preceptor, and in the hurried visits during a short period in a hospital, while attending lectures, and we see all the preparations made on the part of a young physician to combat disease in its multifarious aspects and Protean changes.

Under these circumstances will he dare, inexperienced and unaided, to attempt to meet the exigencies of his position, in which not only his own reputation, but, still more, the life of his patients is at stake; or will he not rather turn for support and counsel to the veterans in the profession, who, in their several works, indicate the dangers in the way and the means by which these have been avoided and overcome? The question ought to admit of but one answer. In such company he gains not only counsel and warning, but often sympathy, encouragement and example, not imposed on him with an air of offensive superiority, but in a spirit of kindness and love of science. Some of these writers may, we grant, express themselves in rough language, some in a quaint and affected style; but we can smile at these blemishes, in consideration of the sterling excellencies of faithful record, judicious reflexions and careful inferences. Strengthened by these aids, the young physician, if he be a regular and candid reader, adding his own observations, at the same time, whenever clinical opportunities are presented to him, will find himself projected, as it were, a decade of years in advance of his age, by his having acquired a knowledge of applied pathology and therapeutics, to meet his own immediate wants and purposes. It may be asked, who are these veteran authors? Have they not become so at the expense of their reputation as useful and successful practitioners; or, in other words, are not the makers of books, to use the language of trade, on that very account less able and willing to compete successfully with their less literary, if not illiterate, professional associates, for business and its emoluments? We proceed at once to answer this question by an appeal to the lives and labors of some of the eminent men who adorn the annals of medicine. We open the appeal by asserting what will be readily proved by evidence soon to be adduced, viz., that good writers must have been good readers and industrious students.

Hippocrates, whom we call the Father of Medicine, was not the first to give it a systematic character, or to keep a record of cases of disease and arrange them under separate heads. He was preceded by a great number of priest physicians, who, like himself, were of the privileged order of Asclepiadæ, or ministers in the temples of Esculapius. From the records kept by them and the votive tablets of grateful patients, in which their diseases and their cures were noted, Hippocrates derived a no small portion of the materials for his works, especially of those on Prognosis, and the Aphorisms; and hence in one sense he may be called a compiler. That he was a student of the past as well as an observer of the present is evident from the large use he made of the library of the Temple of Health at Cnidos, one of the three most eminent medical schools of the time; and that there was an antecedent literature is proved by his treatise on Ancient Medicine. He was trained to a knowledge of dietetics by the perusal of documents preserved in the Asclepion of Cos, one of the many Asclepia erected in various parts of Greece, as receptacles for the sick, and to which libraries were attached. Herodicus was his teacher in applying gymnastics to the cure of disease. Thus prepared by careful study and observation, Hippocrates, who was the son of a physician, "no doubt commenced the practice of his art in the Asclepion of Cos, as his forefathers had done before him,"* but wishing still further to enlarge his field of experience, he visited Thrace, Delos, Thessaly, and many other regions, including Asia Minor, and he practised and probably taught his profession in those places and countries. He thus procured matter for the composition of his great work on Waters, Air, and Places, and of that on Epidemics.

If Hippocrates had contented himself with the routine empiricism of the Asclepion in which his forefathers ministered and practised, and if he had eschewed reading and literature, like some of our self-relying and illiterate physicians, who, amusingly enough, think that they are imitating the sage of Cos by their crudities, where would be our knowledge of ancient Greek medicine, where his posthumous fame, and the lore and the philosophy in example and precept, which he bequeathed to posterity? Lost to us would have been all this, as well as the noble code of ethics contained in his works. Hippocrates, we repeat, was an industrious student, read much, observed much, was an eminent practitioner, and at the same time, a writer of great and, for now more than twenty-two centuries, continued reputation. His learning did not destroy his originality, nor was he less useful and successful in practice because of his eminence as an author.

Galen, the most voluminous of writers, who treated of every branch of medicine and its collateral topics with great copiousness, and wonderful learning and power, was not prevented, on this account, from being a distinguished and an esteemed practitioner of his art, and the favorite physician of four Emperors. Paulus Ægineta, was eminent as a surgeon and accoucheur; a fact, doubtless, of grateful acknowledgment to thousands benefited by his skill and kindness during his life: but what would this have availed posterity, and how would we, at this day, know anything of the practice and precepts of many of his predecessors, were it not for the many volumes which he compiled on these subjects? His learning does not seem to have stood in the way of his becoming as skilful an operator as any mere mechanic in the art of surgery.

The Arabian school of medicine would furnish many examples

* The Genuine Works of Hippocrates, translated from the Greek, with a Preliminary Discourse and Annotations. By Francis Adams, LL. D., Surgeon. Sydenham Society Edition, 2 vols.

in favor of the union which we advocate; but we shall merely mention one of the most distinguished among them, Rhazes, whose medical lore and writings were only equalled by his great tact in detecting the nicest shades of difference in the semeiology of diseases, and by his skill in curing them. In describing the rules for choosing a good physician, the Arabian writer, just named, tells us that the person to be selected ought to combine his own observations with the assiduous reading of good authors, for it is impossible, of one's self, to see all and to experiment on everything; and the learning and experience of a single individual, compared with the learning and experience of all men in all ages, resembles an attenuated rill by the side of a great river.

Coming down to more modern times, we meet with the name of Ambrose Paré, the father of French surgery, who, amid the din of war, in the hours snatched from professional occupations in the camp and on the field of battle, as a military surgeon, found time to educate himself anew, and to make up, by assiduous study, for deficiencies in early life. He was not satisfied with being the first surgeon of his day, nor did he attempt to cover his lack of literary honors by affecting to underrate their value; but he determined to be an Associate of the College of Surgeons of Paris, and asked to be allowed to undergo an examination before that body. After being subjected to this trial, he was made, successively, Bachelor, Licentiate, and Doctor in Surgery. His mind thus stored with abundant knowledge, derived from personal experience and accurate observations, and strengthened by a study of the ancient medical classics, he was prepared to benefit posterity by his numerous writings, which would fill five or six of our common sized octavo volumes.

Baillou, the French Sydenham, and precursor of the celebrated Englishman, by nearly a century, was a professor of belles lettres and commentator on Aristotle, before he obtained the honors of the medical doctorate; but neither these attainments nor his subtle logic interfered, as some of our *practical* brethren might apprehend, with his continued observation of disease, made in the true Hippocratic spirit: nor did his close engagements in this way, and his ever ready services to the poor, prevent his putting on record the fruits of his large experience and study, more especially on epidemics and fixed constitutions of the atmosphere. His works, published from his manuscripts, after his death, amounted to several folio volumes.

Portal gave lectures on anatomy in Montpelier before he had attained the age of manhood, and was an author, by a series of papers on morbid structure and anatomical deviations from nature, when he was twenty five. At this time, also, he edited the *Historia Anatomico-Medica* of Lieutaud. He continued to be a writer during a long life, so as to leave behind him twenty-seven volumes octavo, on a great variety of subjects, including "Medical Anatomy," and the "History of Anatomy and Surgery;" and all this without preventing him from engaging in a large and lucrative practice, including professional attendance on royalty itself, in the person of Louis XVIII. He had the yet higher honor of being the friend and physician of Buffon and D'Alembert. To his other duties Portal added those of a Professor's chair, for a period of forty years, during the greater part of which he taught with untiring zeal and industry. Advanced age neither diminished the vigor of his faculties nor abated his literary and professional ardor. When he was sixty-nine years old he published his work on Apoplexy; when seventy-one, that on the Liver; at eighty, on Dropsy, and at eighty-five, on Epilepsy. He survived until he had passed his ninetieth year.

Among Portal's contemporaries of surgical eminence, the names of Boyer and Larrey occur to us at the moment as having operated and written, we will not say with equal facility, but, at any rate, to such an extent as to show that a large exercise of the pen does not render the hand less steady in the use of the amputating knife, the bistoury and the lithotome.

At the present time, among the eminent men who now figure in the different departments of medicine, in Paris, we may cite M. Velpeau, for the happy union of facile elocution, and copiousness and learning as a writer, with marked ability and success as a general practitioner, and especially as a surgeon and pathologist. Thanks to Drs. Meigs and Mott, who kindly undertook the, to them, grateful office of translating and editing his works on midwifery and surgery, the name and merits of Velpeau have been made familiar to the profession in the United States. The gentlemen first named confirm, in their own professional career, our position, that the being an author does not diminish one's usefulness and reputation in the exercise of practical medicine. To the same purport the name of the late Dr. Dewees offers itself. Faith in his capacity, as an accoucheur and physician, was not shaken in the minds either of the public at large or of his professional brethren, by his being the author of many books on different branches of medicine.

Of the many eminent men in all the departments of medicine whom Italy has produced, we shall content ourselves with the mention of two, whose names would probably first present themselves to the mind of the purely practical physician. They are, Morgagni and Scarpa; companions, for a while, in the relation of preceptor and counsellor on the part of the former; and of admiring pupil and secretary on that of the latter. Some curiosity may naturally be felt, to know what were the early favorite studies, preparations, in a manner, for the subsequent labors of Morgagni, the great anatomist, the prince of anatomical pathologists. Did he amuse himself in boyhood by torturing insects and shooting and then dissecting birds, and by making observations on the viscera of sheep, which had died of the rot? By no means. Under a mother's tender care, this period of life was passed in earnest study, and with such success, that at the age of fourteen years he was made a member of a literary society, called the Academy of Forli, his native town. Nothing hurt by being thus early imbued with learning, he repaired to Bologna to prosecute his medical studies; and there he soon took the place of Valsalva, as Demonstrator of Anatomy, and in a few years after issued the first part of his *Adversaria Anatomica.* For a while he practiced medicine with great success in Forli. How little he was disposed to claim exemption from literary labors, or to be content with the reputation, considerable as that was, acquired in earlier life, we learn from the fact of his being eighty years of age, when his great work *De Sedibus et Causis Morborum* was first published. He survived this event more than nine years, and it was during this period, when his sight failed him, that Scarpa, then prosecuting his medical studies in the University of Padua, became his favorite pupil and his secretary. In the last mentioned capacity Scarpa read the works, and the letters asking the advice of Morgagni, received from different parts of

Europe, and afterwards wrote replies under the dictation of his master.* The amusement in which both participated with great relish, was reading the Latin classics, and especially Plautus, of whom Morgagni was very fond.

Scarpa, himself, was early taught the Humanities, and imbibed a fondness for polite literature, which never left him, and which was the solace of the latter period of his life, when he had retired to a villa not far from Pavia. There he formed a small but a prized collection of paintings and other objects of art, and indulged in his declining years, as he had in his early ones with Morgagni, in reading the Latin classics, among whom Virgil was his favorite and constant companion. With such tastes and culture, we cannot be surprised that Scarpa should have become a prominent laborer in the field of medical literature, acting as he did on the belief, which he held in common with Celsus, that the talent of writing is superior to that of speaking, inasmuch as it perpetuates a knowledge of the different branches of medical science, and assures their progress, at the same time that it assigns to each its appropriate place of honor. His treatises on Diseases of the Eye, Hernia, Club Foot, Aneurism and Ligatures, while they record his clinical experience and evince his literary ability, bear evidence also of his continual reference to healthy and morbid anatomy on which he rests his conclusions. He wrote with equal success on various anatomical subjects, as we may infer from his essays on sight and hearing, and on the cardiac nerves, structure of the bones, ganglial nerves, &c. All this literary labor, alternating with close observation on objects of comparative anatomy and morbid changes of structure, was carried on during the long period in which he was Professor of Anatomy in the University of Pavia, teacher of Clinical Surgery, and engaged in private practice.

We are afraid even to begin an enumeration of the long list of Germans who have combined medical literature with the practice of medicine. Two immediately present themselves to the mind of every English and American reader, although, in fact they were natives of Switzerland, and resided at different pe-

**Maestro*, as we have heard the hospital students in Florence call their clinical chief, when saluting him in the morning.

riods in that country. We refer to the great Haller and to Zimmerman. Haller wrote verses in Latin and German when he was but ten years of age, and up to fifteen years was devoted to literature and poetry. He graduated at eighteen, and at twenty-one, after having travelled in different countries of Europe, he returned to his native city of Berne, and engaged with success in the practice of physic for several years; during part of which time he had charge of a hospital, and delivered lectures on Anatomy. He was not afraid of damaging his professional reputation, at this time, by the publishing of a collection of odes and poetical epistles in German; thus, for the first time wedding anatomy to immortal verse. He gave farther proof of the variety of his accomplishments, during his residence in Berne, by preparing a critical catalogue of all the books in the public library of that city; and arranging after a new method five thousand ancient medals, of which he also prepared a chronological table. Called to Gottingen to occupy in the University the triple chair of anatomy, botany and surgery, he engaged in a course of teaching, and in a series of writings and studies on these branches, and, in a still more enthusiastic manner, on physiology, which have placed him in the foremost rank in medical science. The imagination of the poet offered no impediment to the patient reading, by the botanist, of the works of two hundred and sixty eight writers on botany, whom he quotes in his own great work on the indigenous plants of Switzerland, when describing twenty-five hundred species belonging to that country. But, great and meritorious as were his labors in this way, they were excelled by his immortal Elements of Physiology,* in which this branch was for the first time placed on a scientific basis: nor did he overlook practical medicine and surgery, of which he has collected cases in number to make up several volumes. On his final return to his native city, there to take up his abode for life, he was entrusted with different administrative offices, which he filled with marked abillty; thus showing, in practice, that knowledge of the theory of which he proved himself a master in his treatises on political economy. If it be said that few had the genius and versatile talents of Haller, it ought to be added that quite as few have possessed his zeal for literature and science, his methodical

***Elementa Physiologiæ Corporis Humani.*

habits of study, and above all his unflinching and unwearied industry. Without being ascetics, we are bound to believe that some of his success was due to his habitual temperance, the result of a vow which he made, after a frolic in early life, that he would never more drink wine,—nor did he.

Zimmerman, the pupil of Haller, known to the many by his work on Solitude, and to the profession by his admirable treatises on Experience, and on Dysentery, was a learned man, an elegant writer and an actively engaged practitioner of medicine in his native town of Brug. Cullen thus speaks of the author, in connection with one of the treatises just named: "Zimmerman is the first person who has ever given the true manner of treating dysentery." He resided for some time in Hanover, as physician to the King of England when the latter visited his electorate.

English medical literature furnishes numerous examples of the union of professional acumen and great success in the business of practice with the labors of the desk. The learned Freind was early distinguished, during his collegiate life at Oxford, as the editor of an oration of Eschines against Ctesiphon, and of that of Demosthenes *de Coronâ*, to both of which he added an elegant Latin translation. He, also, studied mathematics and applied himself to anatomy and chemistry, at the same time that he perused with great diligence all the best writers of physic, both ancient and modern. After he had been engaged in practice, he filled the chair of chemistry at Oxford; and, in the following year, was made physician-general to the British army under the Earl of Peterborough in Spain. At a subsequent period, he filled the same post in Flanders, under the duke of Ormond. On his return to London, he edited the first and third books of Hippocrates on Epidemics, and published various essays and letters. It was during his imprisonment in the Tower, for alleged conspiracy against the Hanoverean dynasty, that he began his celebrated work, "The History of Physic, down from the time of Galen to the beginning of the Sixteenth Century." His learning and his studies did not prevent him from being largely engaged in practice, under the great fatigues of which, indeed, his health gave way; and he died at the comparatively early age of fifty-two.

Still more renowned was Freind's intimate associate, Dr. Mead, who, notwithstanding his varied learning, large and lucrative practice, elevated social position, and his possessing a fine collection of paintings and other objects of art, and a large library, is said, by his singular humanity and goodness, to have "conquered even envy itself." His industry must have been equal to his learning, and both were, if possible, surpassed by his generosity, which made him the munificent patron of men of learning and science, both at home and abroad. His extensive correspondence alone, without occupation of his time required by the composition of his different treatises on medical subjects, might have afforded to others, were they similarly situated, an excuse for neglecting the active duties of the profession. But so far was this from being the case that his services were ever promptly rendered to both rich and poor.

Passing to a name of a different order, we meet with John Hunter, the most original genius of the age, whose investigations into the structure of the several classes of animated beings, and the connexion between organization and the display of vital phenomena, were made to illustrate physiology and surgical pathology, who was both a surgeon in hospital and in private practice, and an author of many books. We must all wish that he had been previously more of a reader of books and somewhat conversant with belles lettres, so that he might have been enabled to present his discoveries, and his reflections and conclusions, in a more lucid and consequently more instructive manner.

Abundant learning and highly cultivated taste, with no little proficiency in music and the arts of design, contributed to set off to increased advantage the professional knowledge of John Bell, and to render the usually dry subjects of anatomy and surgery, under his pen, both instructive and entertaining. His eminence as a practical surgeon was undisputed. Charles Bell, with less brilliancy of intellect, but probably of sounder judgment than his brother John, was possessed of similar accomplishments; but he was not, on this account, precluded, as the professional mechanics might, *à priori*, have feared, from making those brilliant discoveries in physiology, which will render his name ever memorable, even if we choose to overlook his Institutes of Surgery and his Anatomy of Expression, and the large share which he had in the joint work by himself and John Bell on Special and Descriptive Anatomy.

The names of Astley Cooper, Lawrence, Brodie, Abernethy and others, will immediately present themselves to the minds of our readers, in support of the point we now affirm, viz.: that great professional eminence, a result of continued professional labor and study, is not incompatible with an indulgence in letters, and the writing of many books. The union implies, it is true, talents and cultivation, and methodical industry of the most persevering kind, which, when displayed, must place their possessor in the high ranks of his professional compeers, and entitle him, at all times, to their gratitude, if not their veneration. We do not wish to be understood as intimating that a learned physician must necessarily be a good practitioner; but we do believe that learning, so far from being an obstacle, will, for reasons already assigned, greatly increase the probability of his becoming one; and that if he fails, owing to mental deficiency or indolence, the failure will be more signal, if not positively disastrous to his patients, by his want of learning.

If what we have written be deemed too long an introduction to our notice of the volume of Dr. La Roche, the blame must rest, in a measure, with the author himself; for, his own mental characteristics and general and medical attainments and experience, joined to practical tact and self-possession in the sick room, chiefly suggested the course which our remarks have taken. They are also in a spirit similar to that of his Introduction, in the form of a letter to his friend, Doctor C. D. Meigs. In it he very happily rebukes the eagerness of youthful sciolists to bring out opinions on the most important questions in medical science and practice, without adequate preparation by previous inquiry, and often in entire ignorance of what had been written on the subject, and fully canvassed and discarded as untenable. He justly remarks, that "the stuff of which medical reformers and leaders in scientific advancements are made is a rare product; that in all parts of the world, and here particularly, more than elsewhere, readiness and smartness have but too often been mistaken for strong powers of thought, and superficial information has taken the place of sound and accurate learning." He believes "that we have, so far as medical literature is concerned, a character, not to uphold merely, but to establish;" and hence, he adds, the necessity of improvements in the various branches of medical science. Among the baleful influences which retard the progress of sound medical literature among us, the author specifies hasty opinions at variance with those deemed by the most enlightened portion of the profession everywhere to be fully established. Sometimes the novelty claimed by its promulgator is such merely from his own ignorance of medical literature. Sometimes, as pointed out in the letter, the cause of the repeated efforts at revolutionizing medicine in some or all of its parts, is due to a craving after notoriety, or a desire on the part of a young writer to see himself in print; and, at times, to a self-esteem which makes a man believe "himself endowed with the faculty of unravelling the most intricate mysteries of the science, and of discovering truths heretofore concealed from the notice of medical investigators since the days of Hippocrates to the present." Another class, of higher attainments and position, and moved by conscientious considerations, err for "the want of a proper balance between the fancy and the judgment;" while others, again, take a devious path, because in the common beaten one they do not find everything they expected. Hasty generalizations from impatience of thorough inquiry, and inability to discover the true relation of cause and effect, are, also, impediments to our reaching the truth. Plagiarism is stigmatized in becoming terms; but with a severity of stricture on our home writers, as if they were the greatest sinners in this respect, which we do not believe to be entirely merited. The sin is great, but we think inquiry would show, that it is not greater on this side of the Atlantic than on the other.

We find that we have reached if not exceeded our prescribed limits in this number of the Journal, and must, therefore, reluctantly postpone the publication of our remarks on the body of this erudite and most valuable work to the next number.

(To be continued.)

*The Pathology and Treatment of Pulmonary Tuberculosis; and on the local medication of Pharyngeal and Laryngeal Diseases, frequently mistaken for, or associated with, Phthisis.* By JOHN HUGHES BENNETT, M. D., F. R. S. E., Professor of the Institutes of Medicine and of Clinical Medicine in the University of Edinburgh, &c. Edinburgh: Sutherland & Knox. 1853.

We have read this brochure of Dr. Bennett with interest and pleasure, as well on account of the views it contains, as of the confidence we have in the judgment and opinions of the author. He endeavors to show,—1st, that tubercular diseases will heal of themselves if the faulty nutrition of the system can be removed; 2ndly, that with this object our efforts should be directed to the digestive, rather than the respiratory system; 3dly, that the kind of abnormal nutrition which exists is dependent on increased assimilation of the albuminous, and diminished assimilation of the fatty portions of the food, and it is mainly in this direction that he calls attention to the pathology of tuberculosis and the therapeutic indications. The author adopts as the basis of his argument the importance of oil in the economy as an adjuvant to the plasticity of albumen, and he supports the doctrine by reference to the constitution of milk, the natural food of young mammiferous animals, and to the egg, which constitutes the source of the tissues of oviparous animals, in both of which, viz., the milk and the egg, oil and albumen are largely found.

The researches of chemists such as Prout and Liebig, point to the same generalization when they assert that carbonized and nitrogenized food are necessary to carry on nutrition, inasmuch as oil is the type of one, and albumen of the other, while the mineral matter is dissolved in both.

The successive changes which occur in the nutritive process for the purpose of assimilation are thus summed up by the author: 1st, introduction into the stomach and alimentary canal of organic matter; 2d, its transformation by the digestive process into albuminous and oily compounds—a chemical process; 3d, the imbibition of these compounds through the mucous membrane in a fluid state, and their union in the termini of the villi and lacteals to form elementary molecules; 4th, the transformation of these, first, into chyle corpuscles; and, secondly, into those of the blood, through the agency of the lymphatic glandular system, which is a vital process. If the fluid thus and subsequently still further elaborated fails in the first stages of the process, the remaining ones must also be interfered with, and a material having feeble plastic powers is deposited in the tissues for their nutrition.

In tuberculosis, the author believes that these elaborating processes are interfered with by a faulty beginning in the digestive function. He quotes Sir Jas. Clark and others in support of his own observations, that phthisis is ushered in with a bad and capricious appetite, a furred tongue, *and unusual acidity** of the stomach and alimentary canal, which symptoms often accompany the disease throughout, becoming more violent towards its close.

By this acidity, the albuminous constituents of the food are rendered easily soluble, and their stomachal digestion uninterfered with; but the case is different as regards the oleaginous matters; they require for their assimilation the action of an alkaline pancreatic secretion, by which, as Bernard has shown, they are emulsified and rendered easily absorbable. In the acid condition referred to as appertaining to phthisis, these secretions are more than neutralized and rendered incapable of emulsifying the fats and preparing them for absorption. Hence an increased amount of albumen enters the blood to the exclusion of the oily matter of the food, whose place is supplied by the absorption of the adipose tissues, causing the emaciation which characterises the disease.

In the mean time albuminous exudations are deposited in the lungs, constituting tubercle. This, in its turn, being deficient in the necessary proportion of fatty matter, is incapable of forming elementary molecules so as to constitute nuclei for further development into cells; they therefore remain abortive and constitute tubercle corpuscles. Thus a local disease is added to the constitutional disorder, and that compound affection is induced called phthisis pulmonalis—consisting of symptoms attributable

*The italics are our own.

partly to the alimentary canal, and partly to the pulmonary organs.

From the above brief exposition of the author's views, the therapeutic indications are evident, and their application will be successful in proportion to the power—1st, of improving the faulty nutrition which is the cause of the exudation assuming a tubercular character; to accomplish which the author urges the use of cod liver oil. 2d, of favoring the absorption of the exudation already poured out, by abstinence from lowering remedies and by counter-irritation. 3d, of preventing the recurrence of fresh exudations by careful hygienic regulations, climate, exercise and good diet.

Under the head of special treatment, there is nothing either striking or novel; and the volume concludes with a chapter on the use of local applications to the pharynx and larynx, in cases mistaken for, or associated with, pulmonary tuberculosis. In this, the well known practice of cauterisation revived by Dr. Green, of New York, is highly commended, and cases illustrative of its efficacy introduced.

To say that we have been pleased with the book would be too faint praise; we have been more than pleased: it has deeply interested us, and has elucidated some points hitherto obscure, and given us a pathology of tuberculosis, on the whole, more satisfactory than any we have met with; and although, as the author remarks, it is probable that some of the details may require modification, it will be found to possess a general harmony with all known facts, while it suggests to the medical practitioner resources, the advantages of which continued experience is daily rendering more evident.

---

*Elements of Human Anatomy*; *General, Descriptive, and Practical.* By T. G. RICHARDSON, M. D., Demonstrator of Anatomy in the Medical Department of the University at Louisville, and one of the Attending Surgeons to the Louisville Marine Hospital. Philada. Lippincott, Grambo, & Co. 1854.

It gives us much pleasure to speak favorably of Dr. Richardson's work. He has united general, descriptive and practical anatomy in the same volume, by which arrangement, the learner just entered upon the study, will be saved the expenditure of much time and labor. The descriptions and dissections are clearly and concisely written. We have been especially pleased with his account of inguinal hernia, which cannot fail, we think, to give the student correct anatomical views of that important affection.

The publishers, Messrs. Lippincott, Grambo & Co., deserve credit for the excellent manner in which the book is printed. The wood-cuts are, generally, well executed.

---

*Ranking's Half-Yearly Abstract, No.* 18. Lindsay & Blakiston, Publishers, Philadelphia.

*Braithwaite's Retrospect of Medicine.* Vol. 28. London.

The deserved popularity of the above works renders any further notice of them unnecessary, than that they are fully equal, as valuable summaries, to their predecessors.

---

*Homœopathy Fairly Represented. A Reply to Professor Simpson's "Homœopathy Misrepresented."* By WM. HENDERSON, M.D., Professor of General Pathology in the University of Edinburgh. Lindsay & Blakiston, Philadelphia.

The above, noticed in connection with Dr. Simpson's work, in our last number, has been received from the publishers.

---

*A Treatise on Venereal Diseases.* By A. VIDAL (DE CASSIS), Surgeon of the Venereal Hospital of Paris, &c. *Translated and Edited by* GEO. C. BLACKMAN, M.D., Fellow of the Royal Medical and Chirurgical Society of London. New York. Samuel S. & W. Wood, 1854.

We regret that our limits have not permitted us to notice M. Vidal's admirable treatise in the present number. We shall merely state that it is rich in practical details, which should, for that reason alone, make it acceptable to the student and practitioner in venereal affections. It will be reviewed in our next number.

